# Correlation between overweightness and the extent of coronary atherosclerosis among the South Caspian population

**DOI:** 10.1186/s12872-020-01534-w

**Published:** 2020-05-29

**Authors:** Maryam Nabati, Mahmood Moosazadeh, Ehsan Soroosh, Hanieh Shiraj, Mahnaneh Gholami, Ali Ghaemian

**Affiliations:** 1 Cardiovascular Research Center, Mazandaran University of Medical Sciences, Sari, Iran; 2 Health Sciences Research Center, Addiction Institute, Mazandaran University of Medical Sciences, Sari, Iran; 3 Department of Cardiology, Golestan University of Medical Sciences, Gorgan, Iran; 4 Mazandaran Heart Center, Artesh BLVD, Sari, Iran

**Keywords:** Body mass index, BMI, coronary artery disease, Waist circumference

## Abstract

**Background:**

Reported effects of obesity on the extent of angiographic coronary artery disease(CAD) have been inconsistent. The present study aimed to investigate the relationships between the indices of obesity and other anthropometric markers with the extent of CAD.

**Methods:**

This study was conducted on 1008 consecutive patients who underwent coronary angiography. Body mass index (BMI), waist circumference (WC), waist-to-hip ratio (WHR), and waist-to-height ratio (WHtR) were separately calculated for each patient. Extent, severity, and complexity of CAD were determined by the Gensini and SYNTAX scores.

**Results:**

According to the results, there was a significant inverse correlation between the SYNTAX score with BMI (r = − 0.110; *P* < 0.001), WC (r = − 0.074; *P* = 0.018), and WHtR (r = − 0.089; *P* = 0.005). Furthermore, a significant inverse correlation was observed between the Gensini score with BMI (r = − 0.090; *P* = 0.004) and WHtR (r = − 0.065; *P* = 0.041). However, the results of multivariate linear regression analysis did not show any association between the SYNTAX and Gensini scores with the indices of obesity and overweight. On the other hand, the patients with an unhealthy WC had a higher prevalence of diabetes mellitus (DM) (*P* = 0.004) and hypertension (HTN) (*P* < 0.001), compared to the patients with healthy values. Coexistence of HTN and DM was more prevalent in subjects with an unhealthy WC and WHR, compared to that in those with healthy values (*P* = 0.002 and *P* = 0.032, respectively).

**Conclusion:**

It seems that the anthropometric indices of obesity are not the predictors of the angiographic severity of CAD. However, they are associated with an increased risk of cardiovascular risk factors and higher risk profile.

## Background

Prevalence of obesity and overweight are increasing worldwide, and they are thought to be linked with the increased risk of several health problems, including type 2 diabetes, hypertension (HTN), coronary artery disease (CAD), and heart failure (HF) [1]. Recent studies have claimed that the measurements of abdominal obesity, including waist circumference (WC), waist-to-height ratio (WHtR), and waist-to-hip ratio (WHR), provide a superior tool for the discrimination between obesity-related cardiometabolic risk and BMI [2, 3].

Reported effects of obesity on the extent of angiographic CAD have been inconsistent [4]. In spite of the unfavorable effects of obesity on cardiovascular risk factors and increased prevalence of CAD among obese patients, some recent studies demonstrated an obesity paradox. It means that overweight and obese patients with known CAD may have a better prognosis than thinner subjects with CAD [5]. The present study aimed to investigate the relationships between BMI, WC, WHtR, WHR, and other anthropometric markers with the severity of CAD.

## Methods

This historical cohort study was performed on 1008 consecutive patients who were admitted to our hospital and underwent coronary angiography due to acute coronary syndrome (ACS), suspected CAD due to chest pain, or the evidence of ischemia on non-invasive studies between September 2017 until October 2018. The study was conducted according to the guidelines of Helsinki Declaration and approved by the local Ethics Committee of our hospital. The patients were excluded from the study if referred for coronary angiography due to indications other than CAD such as valvular or congenital heart diseases, systemic infection, or any other comorbid conditions.

The demographics and medical history, including cardiovascular risk profiles, previous history of coronary artery bypass graft surgery, or percutaneous coronary intervention, were obtained from the subject medical record or face to face questionnaire. The HTN was defined as having a systolic blood pressure ≥ 140 mmHg, diastolic blood pressure ≥ 90 mmHg, or need for antihypertensive medications [6]. Diabetes mellitus (DM) was described according to the guidelines of the American Diabetes Association or need for taking oral hypoglycemic agents or insulin [7].

Family history (FH) of CAD was defined by the diagnosis of the disease in a male first-degree relative < 55 years or female first-degree relative < 65 years [8]. Hyperlipidemia (HLP) was characterized by a serum cholesterol level higher than 5.5 mmol/L and high-density lipoprotein-cholesterol level lower than 1.0 mmol/L in men or lower than 1.1 mmol/L in women [9]. History of smoking was determined by a face to face interview. Hormone replacement therapy was characterized by the combination treatment of estrogen plus progestogen, which was used to treat the symptoms of menopause. The individual’s height (in meters) and weight (in kilograms) were measured, and BMI was calculated as body weight in kilograms divided by height in square metre.

According to the World Health Organization (WHO) classification, the patients in this study were categorized into three groups, namely normal weight (i.e., BMI lower than 25 Kg/m^2^), overweight (i.e., BMI of 25 to lower than 30 Kg/m^2^), and obese (i.e., BMI of 30 Kg/m^2^ and above). The WC was determined as the smallest circumference between the umbilicus and xiphoid process. The patients were then categorized into two groups, including those with a healthy WC (i.e., 88 cm or lower in women and 102 cm or lower in men) and a high risk of WC (i.e., above 88 cm in women and 102 cm in men) [10].

Hip circumference was determined as the largest circumference around the buttocks posteriorly and the symphysis pubis anteriorly. The WHR was then calculated by dividing WC by hip circumference. According to the WHO classification, the subjects were categorized into two groups, including those with a healthy WHR (i.e., 0.9 or lower in men and 0.85 or lower in women) and a high risk of WHR (i.e., 0.91 or higher in men and 0.86 or higher in women). The WHtR was estimated by dividing WC by height. The patients were then categorized into two groups, namely nonobese cases with a WHtR lower than 0.5 and obese patients with a WHtR above 0.5 [11].

Diagnosis of ACS, including non-ST segment elevation myocardial infarction (NSTEMI), ST segment elevation myocardial infarction (STEMI), and unstable angina, was established according to the standards of the European Society of Cardiology (ESC) [12, 13]. The HF was also diagnosed according to the guidelines of the ESC [14]. All patients underwent transthoracic echocardiography within 24 h after hospitalization. Left ventricular ejection fraction (LVEF) was calculated by subtracting left ventricular end-systolic volume (LVESV) from left ventricular end-diastolic volume (LVEDV) divided by LVEDV using the biplane Simpson’s method in apical four- and two-chamber views.

### Angiography

All patients underwent coronary angiography by a Siemens AG (Medical Solutions; Erlangen, Germany). The severity of coronary atherosclerosis was calculated by quantitative coronary angiography method. The angiograms were analyzed by a group of cardiologists. At the end, an experienced interventional cardiologist blinded to the demographic and clinical data of the subjects reviewed the results and approved or corrected them. Significant CAD was defined as lesion ≥50% stenosis of the left main coronary artery and/or ≥ 70% stenosis of other major epicardial coronary arteries or branch vessels [15].

In this study the extent, severity, and complexity of CAD were assessed by the Gensini and SYNTAX scores. The Gensini score was calculated by giving a score to each coronary stenosis [16]. The patients were divided into two groups based on the Gensini score, including those with a low atherosclerotic burden (i.e., Gensini score < 7) and those with a high atherosclerotic burden (i.e., Gensini score ≥ 7) [17].

The total SYNTAX score was determined by summing up the individual scores for each lesion by an algorithm of the SYNTAX score accessible on the website of SYNTAX (http://www.syntaxscore.com) [16]. Again, the subjects were divided into three groups according to the SYNTAX score tertiles, including low: ≤ 21, intermediate: 22–31, and high ≥32 [18]. Calculation of the Gensini and SYNTAX scores were evaluated by repeating the measurements in 20 randomly selected patients within 24 h, and the intra-observer correlation coefficients were reported as 0.94 and 0.93, respectively. An expert interventional cardiologist calculated the scores in all angiograms.

### Statistical analysis

The continuous data were described as mean, standard deviation, as well as minimum and maximum values, and the categorical variables were explained by percentage and frequency. An independent t-test was used to compare the means between two groups in similar continuous. Moreover, dependent variable and analysis of variance were utilized to compare the means between more than two groups. The categorical variables were compared using the Chi-square and Fisher’s exact tests.

Pearson’s correlation coefficient was employed to assess the correlations between different anthropometric variables and extent of coronary atherosclerosis. Furthermore, multivariate linear regression analysis was used to determine the independent relationships between different anthropometric variables and the severity of coronary atherosclerosis. *P*-value ≤0.05 was considered statistically significant. All statistical analyses were performed using SPSS software (version 16).

## Results

A total of 1008 patients, including 606 males and 402 females, were investigated in the present study. The most common CAD risk factor was HTN (635 patients, 63%), followed by having FH of premature CAD (583 patients, 57.8%), DM (391 patients, 38.7%), HLP (234 patients, 23.21%), and cigarette smoking (157 patients, 15.57%). Mean scores of age for male and female patients were 56.85 ± 11.41 and 57.21 ± 10.77 (26–90) years, respectively (*P* = 0.194). The mean BMI values were 27.49 ± 4.07 and 29.97 ± 5.30 kg/m^2^ for male and female patients, respectively (*P* < 0.001).

The mean SYNTAX score was higher in men, compared to that in women (9.29 ± 10.34 and 6.98 ± 9.97, respectively; P < 0.001). Furthermore, the elderly patients aged ≥70 years had higher SYNTAX scores, compared to the subjects under 50 years of age (12.04 ± 11.56 and 4.13 ± 7.20, respectively; *P* < 0.001). The SYNTAX score was significantly higher in diabetics in comparison to that in nondiabetics (9.79 ± 10.60 and 7.47 ± 9.92, respectively; P < 0.001). In addition, the SYNTAX score was significantly higher in hypertensives than that in normotensive patients (9.25 ± 10.59 and 6.81 ± 9.43, respectively; *P* < 0.001). Table 1 tabulates the clinical and demographic variables of the study population based on the SYNTAX score tertiles.

**Table 1:** Anthropometric variables of the study population as divided by the tertiles of the SYNTAX score

Variables		Total	SYNTAX Score Group; n(%)				Mean ± SD	***P***-Value
			0–21	22–31	> = 32	P-value		
Gender (frequency, percentile)	Male	606	513(84.7%)	69(11.4%)	24(4.0%)	0.250	9.29 ± 10.34	< 0.001
	Female	402	354(88.1%)	33(8.2%)	15(3.7%)		6.98 ± 9.97	
Age groups (years)	< 40	51	51(100.0%)	0(.0%)	0(.0%)	< 0.001	2.09 ± 3.82	< 0.001
	40–49	175	164(93.7%)	9(5.1%)	2(1.1%)		4.72 ± 7.82	
	50–59	385	328(85.2%)	46(11.9%)	11(2.9%)		8.51 ± 9.70	
	60–69	261	217(83.1%)	31(11.9%)	13(5.0%)		9.91 ± 11.34	
	> = 70	136	107(78.7%)	16(11.8%)	13(9.6%)		12.04 ± 11.56	
HTN (frequency, percentile)	Yes	635	536(84.4%)	69(10.9%)	30(4.7%)	0.083	9.2510.59	< 0.001
	No	372	331(89.0%)	32(8.6%)	9(2.4%)		6.81 ± 9.43	
DM (frequency, percentile)	Yes-Insulin	108	86(79.6%)	18(16.7%)	4(3.7%)	0.015	10.60 ± 10.53	0.001
	noninsulin	283	234(82.7%)	37(13.1%)	12(4.2%)		9.48 ± 10.63	
	no	617	547(88.7%)	47(7.6%)	23(3.7%)		7.47 ± 9.92	
DM (frequency, percentile)	Yes	391	320(81.8%)	55(14.1%)	16(4.1%)	0.004	9.79 ± 10.60	< 0.001
	No	617	547(88.7%)	47(7.6%)	23(3.7%)		7.47 ± 9.92	
CABG or PCI (frequency, percentile)	Yes	111	100(90.1%)	8(7.2%)	3(2.7%)	0.422	7.49 ± 8.85	0.344
	No	833	755(85.5%)	92(10.4%)	36(4.1%)		8.47 ± 10.42	
ACS or HF (frequency, percentile)	Yes	439	366(83.4%)	53(12.1%)	20(4.6%)	0.105	9.58 ± 10.62	0.001
	No	569	501(88.0%)	49(8.6%)	19(3.3%)		7.43 ± 9.86	
FH of premature CAD (frequency, percentile)	Yes	583	502(86.1%)	61(10.5%)	20(3.4%)	0.654	7.94 ± 10.009	0.122
	No	425	365(85.9%)	41(9.6%)	19(4.5%)		8.95 ± 10.56	
Smoker (frequency, percentile)	Yes	157	138(87.9%)	16(10.2%)	3(1.9%)	0.381	8.60 ± 9.16	0.753
	No	850	728(85.6%)	86(10.1%)	36(4.2%)		8.32 ± 10.44	
Menopause (frequency, percentile)	Yes	289	247(85.5%)	28(9.7%)	14(4.8%)	0.031	8.22 ± 10.64	< 0.001
	No	113	107(94.7%)	5(4.4%)	1(.9%)		3.82 ± 7.10	
HRT (frequency, percentile)	Yes	78	71(91.0%)	5(6.4%)	2(2.6%)	0.413	5.55 ± 8.97	0.012
	No	930	796(85.6%)	97(10.4%)	37(4.0%)		8.60 ± 10.32	
MI (frequency, percentile)	Yes	73	53(72.6%)	13(17.8%)	7(9.6%)	0.002	13.06 ± 11.58	< 0.001
	No	935	814(87.1%)	89(9.5%)	32(3.4%)		8.00 ± 10.05	
ACS (frequency, percentile)	Yes	392	330(84.2%)	44(11.2%)	18(4.6%)	0.389	9.45 ± 10.48	0.007
	No	616	537(87.2%)	58(9.4%)	21(3.4%)		7.68 ± 10.04	
Type of presentation (frequency, percentile)	STEMI	77	61(79.2%)	11(14.3%)	5(6.5%)	0.001	11.79 ± 10.07	< 0.001
	Non STEMI	51	36(70.6%)	10(19.6%)	5(9.8%)		14.67 ± 11.92	
	Unstable Angina	35	25 (71.4%)	8(22.9%)	2(5.7%)		13.05 ± 11.09	
	Stable Angina	707	622(88.0%)	60(8.5%)	25(3.5%)		7.71 ± 9.99	
	Others	138	123(89.1%)	13(9.4%)	2(1.4%)		6.31 ± 9.26	
WC (cm)	< 102 or < 88	367	311(84.7%)	39(10.6%)	17(4.6%)	0.570	9.52 ± 10.22	0.007
	> = 102 or > =88	641	556(86.7%)	63(9.8%)	22(3.4%)		7.71 ± 10.21	
W/HR	<=0.9 or < =0.85	28	26(92.9%)	2(7.1%)	0(.0%)	0.467	6.14 ± 7.81	0.244
	> 0.9 or > 0.85	980	841(85.8%)	100(10.2%)	39(4.0%)		8.43 ± 10.31	
W/HtR	< 0.5	60	51(85.0%)	4(6.7%)	5(8.3%)	0.132	10.01 ± 10.68	0.201
	> = 0.5	948	816(86.1%)	98(10.3%)	34(3.6%)		8.26 ± 10.22	
BMI (kg/m^2^)	< 25	226	191(84.5%)	23(10.2%)	12(5.3%)	0.111	9.64 ± 10.41	0.004
	25–29	465	392(84.3%)	57(12.3%)	16(3.4%)		8.77 ± 10.17	
	> = 30	317	284(89.6%)	22(6.9%)	11(3.5%)		6.87 ± 10.10	
HLP (frequency, percentile)	Yes	234	185(79.1%)	35(15.0%)	14(6.0%)	0.002	10.43 ± 11.27	< 0.001
	No	774	682(88.1%)	67(8.7%)	25(3.2%)		7.74 ± 9.84	

*HTN* Hypertension, *DM* Diabetes mellitus, *CABG* Coronary artery bypass graft, *PCI* Percutaneous coronary intervention, *ACS* Acute coronary syndrome, *HF* Heart failure, *FH* Family history, *HRT* Hormone replacement therapy, *MI* Myocardial infarction, *STEMI* ST-elevation myocardial infarction, *Non STEMI* Non ST-elevation myocardial infarction, *WC* Waist circumference, *W/HR* Waist to hip ratio, *W/HtR* Waist to height ratio, *BMI* Body mass index, *HLP* Hyperlipidemia, *CAD* Coronary artery disease

**Fig. 1:**
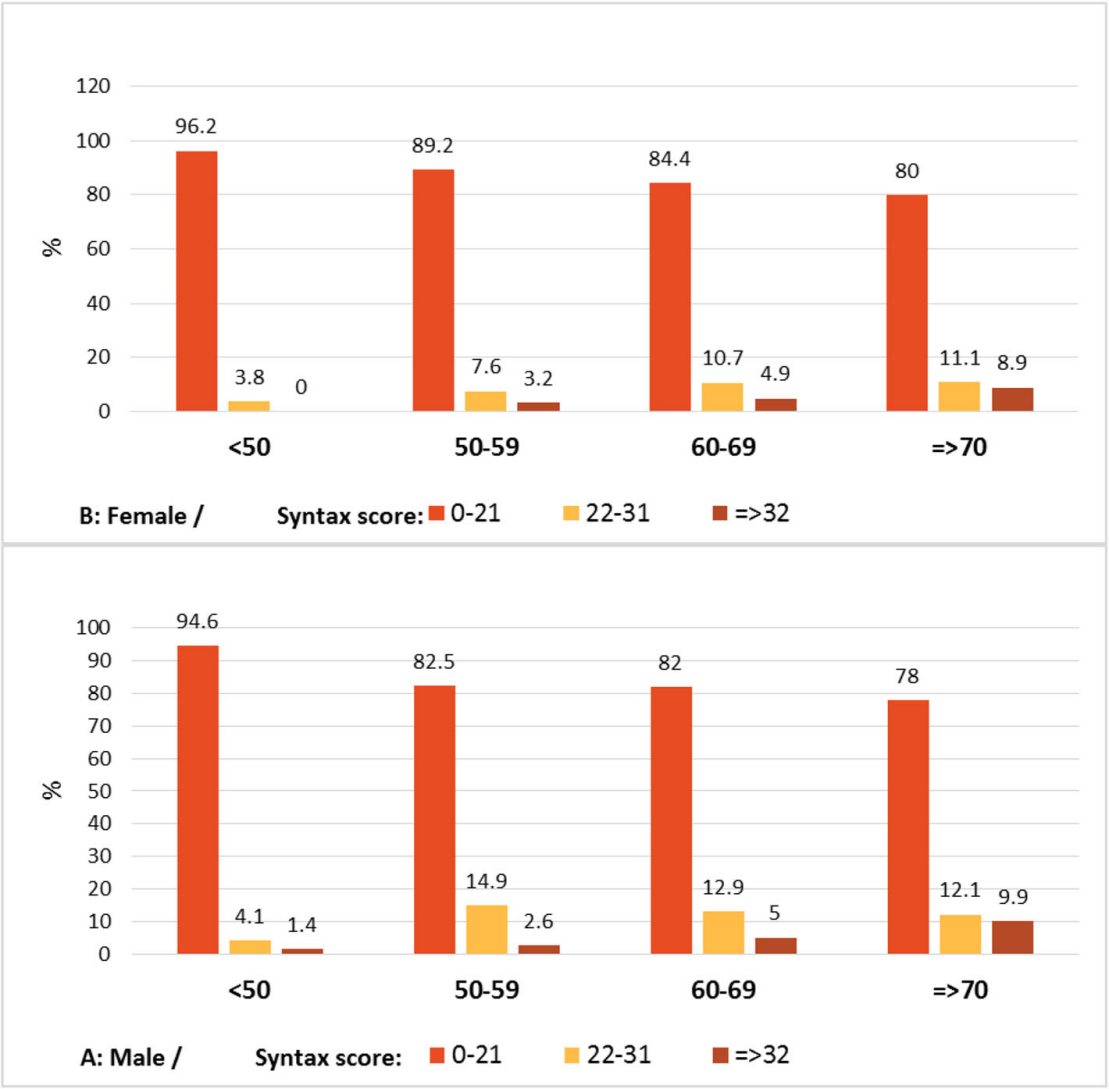
The severity of CAD expressed by SYNTAX score in different age groups categorized by sex.

**Fig. 2:**
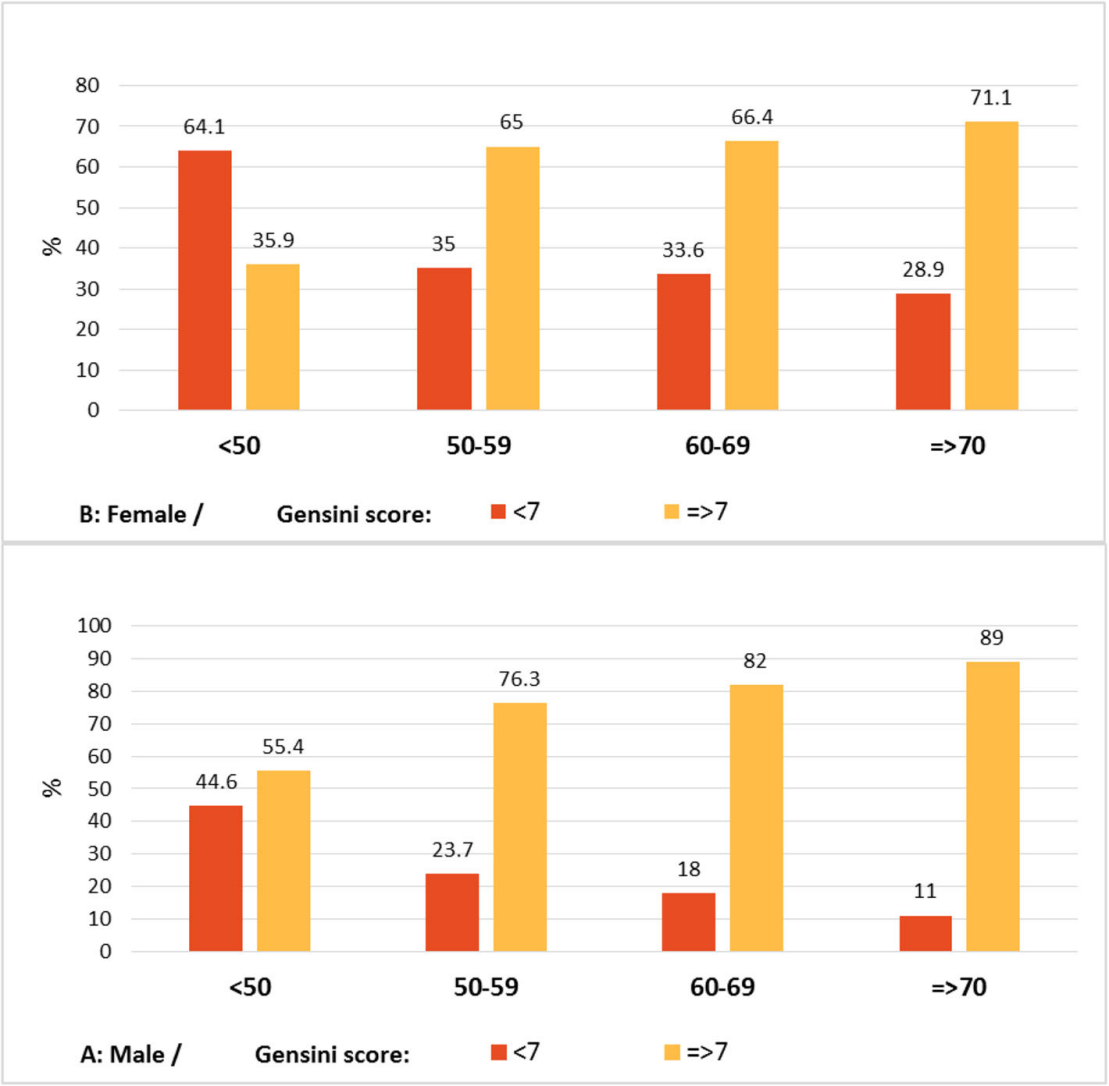
The severity of CAD expressed by gensini score in different age groups categorized by sex

Among the patients with the SYNTAX score < 21, male subjects had a higher prevalence of the scores between 9.1 and 21, compared to female patients (26.9% vs. 16.4%; Table 2; *P* < 0.001). Regarding the Gensini score, it was significantly higher in men than that in women (28.13 ± 28.21 vs. 21.32 ± 27.86, respectively; P < 0.001), higher in hypertensives than that in normotensive subjects (27.64 ± 28.91 vs. 21.56 ± 26.70, respectively; *P* = 0.01), and higher in hyperlipidemics than that in normolipidemics (31.11 ± 31.65 vs. 23.71 ± 26.93; Table 3; *P* < 0.001). Severity of CAD expressed by the SYNTAX and Gensini scores in different age groups categorized by sex is represented in Figs. 1 and 2.

**Table 2:** The distribution of anthropometric variables in patients with low SYNTAX score (< 21)

Variables		Total	Syntax Score < 21; n(%)			
			0	0.01–9	9.1–21	***P***-value
Gender (frequency, percentile)	Male	513	200(39.0%)	175(34.1%)	138(26.9%)	< 0.001
	Female	354	194(54.8%)	102(28.8%)	58(16.4%)	
Age groups (years)	< 40	51	35(68.6%)	11(21.6%)	5(9.8%)	< 0.001
	40–49	164	102(62.2%)	39(23.8%)	23(14.0%)	
	50–59	328	139(42.4%)	114(34.8%)	75(22.9%)	
	60–69	217	88(40.6%)	70(32.3%)	59(27.2%)	
	> = 70	107	30(28.0%)	43(40.2%)	34(31.8%)	
HTN frequency, percentile)	Yes	536	219(40.9%)	183(34.1%)	134(25.0%)	0.002
	No	331	175(52.9%)	94(28.4%)	62(18.7%)	
DM (frequency, percentile)	Yes-Insulin	86	31(36.0%)	27(31.4%)	28(32.6%)	0.031
	-noninsulin	234	98(41.9%)	75(32.1%)	61(26.1%)	
	no	547	265(48.4%)	175(32.0%)	107(19.6%)	
DM (frequency, percentile)	Yes	320	129(40.3%)	102(31.9%)	89(27.8%)	0.011
	No	547	265(48.4%)	175(32.0%)	107(19.6%)	
CABG or PCI frequency, percentile)	Yes	100	36(36.0%)	45(45.0%)	19(19.0%)	0.011
	No	755	353(46.8%)	228(30.2%)	174(23.0%)	
ACS or HF frequency, percentile)	Yes	366	134(36.6%)	143(39.1%)	89(24.3%)	< 0.001
	No	501	260(51.9%)	134(26.7%)	107(21.4%)	
FH of premature CAD frequency, percentile)	Yes	502	240(47.8%)	159(31.7%)	103(20.5%)	0.150
	No	365	154(42.2%)	118(32.3%)	93(25.5%)	
Smoker frequency, percentile)	Yes	138	49(35.5%)	48(34.8%)	41(29.7%)	0.021
	No	728	345(47.4%)	229(31.5%)	154(21.2%)	
Menopause frequency, percentile)	Yes	247	120(48.6%)	79(32.0%)	48(19.4%)	0.001
	No	107	74(69.2%)	23(21.5%)	10(9.3%)	
HRT frequency, percentile)	Yes	71	44(62.0%)	18(25.4%)	9(12.7%)	0.011
	No	796	350(44.0%)	259(32.5%)	187(23.5%)	
MI (frequency, percentile)	Yes	53	9(17.0%)	28(52.8%)	16(30.2%)	< 0.001
	No	814	385(47.3%)	249(30.6%)	180(22.1%)	
ACS (frequency, percentile)	Yes	330	122(37.0%)	126(38.2%)	82(24.8%)	< 0.001
	No	537	272(50.7%)	151(28.1%)	114(21.2%)	
Type of presentation (frequency, percentile)	STEMI	61	9(14.8%)	32(52.5%)	20(32.8%)	< 0.001
	NSTEMI	36	6(16.7%)	16(44.4%)	14(38.9%)	
	Unstable Angina	25	8(32.0%)	8(32.0%)	9(36.0%)	
	Stable Angina	622	297(47.7%)	192(30.9%)	133(21.4%)	
	Others	123	74(60.2%)	29(23.6%)	20(16.3%)	
WC (cm)	< 102 or < 88	311	114(36.7%)	113(36.3%)	84(27.0%)	< 0.001
	> = 102 or > =88	556	280(50.4%)	164(29.5%)	112(20.1%)	
W/HR	<=0.9 or < =0.85	26	14(53.8%)	5(19.2%)	7(26.9%)	0.369
	> 0.9 or > 0.85	841	380(45.2%)	272(32.3%)	189(22.5%)	
W/HtR	< 0.5	51	19(37.3%)	17(33.3%)	15(29.4%)	0.379
	> = 0.5	816	375(46.0%)	260(31.9%)	181(22.2%)	
BMI (kg/m^2^)	< 25	191	70(36.6%)	67(35.1%)	54(28.3%)	0.004
	25–29	392	173(44.1%)	125(31.9%)	94(24.0%)	
	> = 30	284	151(53.2%)	85(29.9%)	48(16.9%)	
HLP (frequency, percentile)	Yes	185	80(43.2%)	54(29.2%)	51(27.6%)	0.185
	No	682	314(46.0%)	223(32.7%)	145(21.3%)	

*HTN* Hypertension, *DM* Diabetes mellitus, *CABG* Coronary artery bypass graft, *PCI* Percutaneous coronary intervention, *ACS* Acute coronary syndrome, *HF* Heart failure, *FH* Family history, *HRT* Hormone replacement therapy, *MI* Myocardial infarction, *STEMI* ST-elevation myocardial infarction, *NSTEMI* Non ST-elevation myocardial infarction, *WC* Waist circumference, *W/HR* Waist to hip ratio, *W/HtR* Waist to height ratio, *BMI* Body mass index, *HLP* Hyperlipidemia, *CAD* Coronary artery disease

**Table 3:** Anthropometric variables of the study population as divided by Gensini score groups

Variables		Total	Gensini score; n(%)			Mean ± SD	***P***-Value
			< 7	> = 7	***P***-value		
Gender (frequency, percentile)	Male	606	155(25.6%)	451(74.4%)	< 0.001	28.13 ± 28.21	< 0.001
	Female	402	159(39.6%)	243(60.4%)		21.35 ± 27.86	
Age groups (years)	< 40	51	33(64.7%)	18(35.3%)	< 0.001	6.91 ± 10.48	< 0.001
	40–49	175	83(47.4%)	92(52.6%)		14.97 ± 19.16	
	50–59	385	109(28.3%)	276(71.7%)		27.45 ± 29.35	
	60–69	261	66(25.3%)	195(74.7%)		27.68 ± 29.61	
	> = 70	136	23(16.9%)	113(83.1%)		35.80 ± 30.03	
HTN (frequency, percentile)	Yes	635	169(26.6%)	466(73.4%)	< 0.001	27.64 ± 28.91	0.001
	No	372	145(39.0%)	227(61.0%)		21.56 ± 26.70	
DM (frequency, percentile)	Yes-Insulin	108	26(24.1%)	82(75.9%)	0.009	31.85 ± 31.30	< 0.001
	noninsulin	283	74(26.1%)	209(73.9%)		29.65 ± 31.18	
	no	617	214(34.7%)	403(65.3%)		22.37 ± 25.79	
DM (frequency, percentile)	Yes	391	100(25.6%)	291(74.4%)	0.002	30.26 ± 31.19	< 0.001
	No	617	214(34.7%)	403(65.3%)		22.37 ± 25.79	
CABG or PCI	Yes	111	27(24.3%)	84(75.7%)	0.098	25.69 ± 30.29	0.881
	No	883	283(32.0%)	600(68.0%)		25.26 ± 27.82	
ACS or HF	Yes	439	107(24.4%)	332(75.6%)	< 0.001	29.46 ± 30.54	0.000
	No	569	207(36.4%)	362(63.6%)		22.32 ± 25.97	
FH of premature CAD (frequency, percentile)	Yes	583	188(32.2%)	395(67.8%)	0.379	24.86 ± 28.71	0.456
	No	425	126(29.6%)	299(70.4%)		26.20 ± 27.64	
Smoker (frequency, percentile)	Yes	157	37(23.6%)	120(76.4%)	0.025	26.63 ± 25.69	.554
	No	850	277(32.6%)	573(67.4%)		25.18 ± 28.72	
Menopause (frequency, percentile)	Yes	289	96(33.2%)	193(66.8%)	< 0.001	24.75 ± 30.37	< 0.001
	No	113	63(55.8%)	50(44.2%)		12.66 ± 17.39	
HRT (frequency, percentile)	Yes	78	34(43.6%)	44(56.4%)	0.014	16.58 ± 20.28	0.004
	No	930	280(30.1%)	650(69.9%)		26.17 ± 28.71	
MI (frequency, percentile)	Yes	73	8(11.0%)	65(89.0%)	< 0.001	40.80 ± 32.14	< 0.001
	No	935	306(32.7%)	629(67.3%)		24.23 ± 27.59	
ACS (frequency, percentile)	Yes	392	95(24.2%)	297(75.8%)	< 0.001	29.00 ± 29.88	0.001
	No	616	219(35.6%)	397(64.4%)		23.15 ± 26.95	
Type of presentation (frequency, percentile)	STEMI	77	9(11.7%)	68(88.3%)	< 0.001	33.51 ± 27.41	< 0.001\
	NSTEMI	51	6(11.8%)	45(88.2%)		41.15 ± 35.58	
	Unstable Angina	35	6(17.1%)	29(82.9%)		30.22 ± 23.55	
	Stable Angina	707	237(33.5%)	470(66.5%)		24.05 ± 28.07	
	Others	138	56(40.6%)	82(59.4%)		20.94 ± 25.05	
WC (cm)	< 102 or < 88	367	89(24.3%)	278(75.7%)	< 0.001	27.73 ± 26.98	0.050
	> = 102 or > =88	641	225(35.1%)	416(64.9%)		24.11 ± 28.90	
W/HR	<=0.9 or < =0.85	28	10(35.7%)	18(64.3%)	0.597	15.92 ± 15.11	0.071
	> 0.9 or > 0.85	980	304(31.0%)	676(69.0%)		25.70 ± 28.50	
W/HtR	< 0.5	60	14(23.3%)	46(76.7%)	0.178	25.62 ± 24.34	0.956
	> = 0.5	948	300(31.6%)	648(68.4%)		25.42 ± 28.50	
BMI (kg/m^2^)	< 25	226	53(23.5%)	173(76.5%)	< 0.001	27.15 ± 27.00	0.015
	25–29	465	137(29.5%)	328(70.5%)		27.18 ± 28.65	
	> = 30	317	124(39.1%)	193(60.9%)		21.62 ± 28.26	
HLP ((frequency, percentile)	Yes	234	57(24.4%)	177(75.6%)	0.010	31.11 ± 31.65	< 0.001
	No	774	257(33.2%)	517(66.8%)		23.71 ± 26.93	

*HTN* Hypertension, *DM* Diabetes mellitus, *CABG* Coronary artery bypass graft, *PCI* Percutaneous coronary intervention, *ACS* Acute coronary syndrome, *HF* Heart failure, *FH* Family history, *HRT* Hormone replacement therapy, *MI* Myocardial infarction, *STEMI* ST-elevation myocardial infarction, *NSTEMI* Non ST-elevation myocardial infarction, *WC* Waist circumference, *W/HR* Waist to hip ratio, *W/HtR* Waist to height ratio, *BMI* Body mass index, *HLP* Hyperlipidemia, *CAD* Coronary artery disease

Pearson’s correlation coefficient was used to assess the association between the different anthropometric variables and Gensini and SYNTAX scores. According to the results, there was a significant direct association between the SYNTAX score and age (r = 0.250; *P* < 0.001) and a significant inverse correlation between the SYNTAX score with BMI (r = − 0.110; *P* < 0.001), LVEF (r = − 0.248; *P* < 0.001), WC (r = − 0.074; *P* = 0.018), WHR (r = − 0.009; *P* = 0.780), and WHtR (r = − 0.089; *P* = 0.005). Furthermore, there was a significant direct association between the Gensini score and age (r = 0.240; *P* < 0.001) and a significant inverse correlation between the Gensini score with BMI (r = − 0.090; *P* = 0.004), LVEF (r = − 0.253; *P* < 0.001), and WHtR (r = − 0.065; *P* = 0.041).

Then, multivariate linear regression analysis was conducted to modulate the confounding effects of other variables on the SYNTAX and Gensini scores (Tables 4 and 5). These analyses did not show any association between the SYNTAX and Gensini scores with the indices of obesity and overweightness.

**Table 4:** Multivariate predictors of atherosclerosis severity (SYNTAX score)

Variables		β	***P***-value
Gender	Male	–	–
	Female	− 0.09	0.020
HTN	Yes	0.04	0.146
	No	–	–
DM	Yes	0.09	0.002
	No	–	–
ACS or HF	Yes	−0.01	0.722
	No	–	–
HRT	Yes	−0.02	0.484
	No	–	–
MI	Yes	0.07	0.045
	No	–	–
Type of presentation	STEMI	0.06	0.146
	NSTEMI	0.13	< 0.001
	Unstable Angina	0.07	0.033
	Stable Angina	0.03	0.379
	Others	–	–
HLP	Yes	0.14	< 0.001
	No	–	–
Age		0.19	< 0.001
LVEF		−0.15	< 0.001
WC		0.003	0.970
WHtR		0.01	0.897
BMI		−0.06	0.290

*HTN* Hypertension, *DM* Diabetes mellitus, *ACS* Acute coronary syndrome, *HF* Heart failure, *HRT* Hormone replacement therapy, *MI* Myocardial infarction, *STEMI* ST-elevation myocardial infarction, *NSTEMI* Non ST-elevation myocardial infarction, *WC* Waist circumference, *WHtR* Waist to height ratio, *BMI*: Body mass index, *HLP* Hyperlipidemia, *LVEF* Left ventricular ejection fraction

**Table 5:** Multivariate predictors of atherosclerosis severity (Gensini score)

Variables		β	*P*-value
Gender	Male	–	–
	Female	− 0.11	0.010
HTN	Yes	0.03	0.343
	No	–	–
DM	Yes	0.12	< 0.001
	No	–	–
ACS or HF	Yes	0.01	0.691
	No	–	–
HRT	Yes	−0.03	0.300
	No	–	–
MI	Yes	0.10	0.003
	No	–	–
Type of presentation	STEMI	0.008	0.841
	NSTEMI	0.11	0.002
	Unstable Angina	0.009	0.760
	Stable Angina	0.02	0.618
	Others	–	–
HLP	Yes	0.14	< 0.001
	No	–	–
Age		0.18	< 0.001
EF		−0.16	< 0.001
WC		0.02	0.809
WHtR		0.03	0.779
BMI		−0.07	0.212

*HTN* Hypertension, *DM* Diabetes mellitus, *ACS* Acute coronary syndrome, *HF* Heart failure, *HRT* Hormone replacement therapy, *MI* Myocardial infarction, *STEMI* ST-elevation myocardial infarction, *NSTEMI* Non ST-elevation myocardial infarction, *WC* Waist circumference, *W/HtR* Waist to height ratio, *BMI* Body mass index, *HLP* Hyperlipidemia, *LVEF* Left ventricular ejection fraction

In the present study, the prevalence rate of DM was reported as 38.8%. The patients with an unhealthy WC had a higher prevalence of DM, compared to the subjects with a healthy WC (42.1% vs. 33%; *P* = 0.004). In addition, the prevalence rate of HTN was reported to be 63%. The patients with an unhealthy WC had a higher prevalence of HTN, compared to the subjects with a healthy WC (68.6% vs. 53.1%; *P* < 0.001). Moreover, the obese and overweight patients had a higher prevalence of HTN, compared to the subjects with a normal weight (68.5% vs. 58.4 and 61.5% vs. 58.4%, respectively; *P* = 0.038). Coexistent HTN and DM were observed in 290 cases (28.8%), and this coexistence was more prevalent in patients with an unhealthy WC and WHR, compared to that in subjects with healthy values (32.1% vs. 22.9%, *P* = 0.002, and 29.3% vs. 10.7%, *P* = 0.032, respectively) (Table 6).

**Table 6:** Comparison of anthropometric indices among patients with diabetes, blood pressure and coexistence of DM and HTN

Variables		Total	DM; n (%)		***P***	HTN; n (%)		P	DM and HTN; n (%)		***P***
			Yes = 391	No = 617		Yes = 635	No = 373		Yes = 290	No = 718	
WC	< 102 or < 88	367	121(33)	246(67)	0.004	195(53.1)	172(46.9)	< 0.001	84(22.9)	283(77.1)	0.002
	> = 102 or > =88	641	270(42.1)	371(57.9)		440(68.6)	201(31.4)		206(32.1)	435(67.9)	
W/HR	<=0.9 or < =0.85	28	8(28.6)	20(71.4)	0.260	11(39.3)	17(60.7)	0.008	3(10.7)	25(89.3)	0.032
	> 0.9 or > 0.85	980	383(39.1)	597(60.9)		624(63.7)	356(36.3)		287(29.3)	693(70.7)	
W/HtR	< 0.5	60	24(40)	36(60)	0.843	33(55)	27(45)	0.186	17(28.3)	43(71.7)	0.939
	> = 0.5	948	367(38.7)	581(61.3)		602(63.5)	346(36.5)		273(28.8)	675(71.2)	
BMI	< 25	226	76(33.6)	150(66.4)	0.194	132(58.4)	94(41.6)	0.038	54(23.9)	172(76.1)	0.183
	25–29	465	188(40.4)	277(59.6)		286(61.5)	179(38.5)		141(30.3)	324(69.7)	
	> = 30	317	127(40.1)	190(59.9)		217(68.5)	100(31.5)		95(30)	222(70)	

*HTN* Hypertension, *DM* Diabetes mellitus, *WC* Waist circumference, *W/HR* Waist to hip ratio, *W/HtR* Waist to height ratio, *BMI* Body mass index

## Discussion

In this study, it was observed that the patients with higher SYNTAX and Gensini scores were older with a higher prevalence of DM, HTN, and HLP, compared to those with lower scores. The subjects with higher SYNTAX and Gensini scores also had a lower WC and BMI and showed a higher probability of undergoing coronary angiography due to ACS or HF than the patients with lower scores. A significant direct correlation was also observed between the Gensini and SYNTAX scores with age and an inverse correlation with LVEF, BMI, WC, and WHtR. After the modulation of the confounding effects of other variables, male gender, age, DM, HLP, and NSTEMI appeared to predict the extent of coronary atherosclerosis. However, BMI and other indices of abdominal obesity did not show to be the predictors of severe CAD.

Based on the literature, it was shown that the anthropometric indices of obesity and overweightness have been associated with higher cardiometabolic risk and were reported to have good predictive values for assessing the probability of CAD [2]. Although BMI as an index of obesity is linked to the increased risk of cardiovascular disease, it seems that the pattern of body fat distribution is a more important determinant of risk than BMI [19].

The WHtR is an effective abdominal obesity index that has been proved to have a robust association with cardiovascular risk factors in Asian population and predicts the risk of diabetes and coronary heart diseases in the general population [17]. Due to the adverse effects of obesity on cardiovascular risk factors, as well as the structure and function of the heart, the prevalence of all cardiovascular diseases increased in the setting of obesity. However, many studies using the various measurements of obesity demonstrated an obesity paradox in patients with CAD with good prognosis among overweight and obese patients with CAD [5].

Rubinshtein et al. evaluated the correlation between BMI, extent of CAD, and prevalence of high-risk coronary anatomy in 928 patients who underwent coronary angiography. It was observed that obese subjects were younger with a lower prevalence of high-risk coronary anatomy. Obese patients were probably referred for angiography earlier than nonobese cases, and this may explain the obesity paradox in these subjects [20]. In another study, the BMI was compared among 842 patients with and without angiographic CAD who underwent coronary angiography. The subjects with coronary stenosis > 50% were less likely to be obese and more likely to be at ideal body weight, compared to the patients with a lower degree of coronary stenosis [4].

Again, another cohort study evaluated 1299 patients who had undergone coronary angiography to determine if there was a significant correlation between BMI with the extent of coronary atherosclerosis, coronary events, and mortality. The Gensini score was used to determine the burden of coronary atherosclerosis. Overweight and obese subjects had a higher prevalence of HLP, HTN, and DM, compared to normal-weight patients; however, BMI was not significantly associated with a higher extent of coronary atherosclerosis. Mortality due to cardiac events was not different between groups. However, obese and overweight patients had a higher incidence of coronary events, compared to normal-weight patients [21].. This finding is similar to the results in the present study that the various indices of obesity were not the predictors of the extent of coronary atherosclerosis in spite of being associated with cardiovascular risk factors, such as aging, HLP, and DM. Higher incidence of coronary events in obese subjects is probably due to associated cardiovascular risk factors, impaired endothelial function, and inflammation [21]. Paradoxical association of obesity with a lower burden of coronary atherosclerosis may be partly due to the limitations of noninvasive studies to accurately diagnose the severity of CAD in obese patients resulting in the referral of the subjects without disease for angiography at an earlier time [22].

On the other hand, a low BMI may indicate a low level of serum cholesterol, triglyceride, total fat-free mass, and other anthropometric indices, such as a small thigh circumference, which has been related to the total mortality [23]. Severe CAD is associated with a lower LVEF, compared to single-vessel or two-vessel disease. Reduced LVEF is considered an important parameter of identifying high-risk patients, who are most likely to benefit from a more aggressive treatment [24]. This finding is consistent with the results of the present study that the patients with a more extensive CAD had a lower LVEF, compared to the subjects with a lower level of extensive disease.

In the present study, the subjects with NSTEMI had higher SYNTAX and Gensini scores in comparison to the patients with STEMI. This result is consistent with the findings of previous studies indicating that the majority of culprit lesions in STEMI were associated with a less complex structure, compared to those causing NSTEMI [25].

### Limitation

In our study, echocardiographic assessment of LVEF was not performed independently by two researchers. However, evaluation of echocardiographic data was not the main purpose of this study. Also, our study was cross-sectional and the duration of overweightness which may influence the results was not included in the study. Thus, this study could not show any causal relationship between obesity and the severity of CAD.

## Conclusion

In conclusion, it seems that the anthropometric indices of obesity are not the predictors of the severity of CAD. However, the anthropometric indices of obesity are associated with the increased risk of cardiovascular risk factors and high-risk profile.

## Data Availability

The datasets used and/or analysed during the current study are available from the corresponding author on reasonable request.

## Abbreviations

CAD: Coronary artery disease
BMI: Body mass index
WC: Waist circumference
WHR: Waist-to-hip ratio
WHtR: Waist-to-height ratio
DM: Diabetes mellitus
HTN: Hypertension
HR: Heart failure
WHO: World Health Organization
ESC: European society of cardiology
LVEF: Left ventricular ejection fraction
LVESV: Left ventricular end-systolic volume
LVEDV: Left ventricular end-diastolic volume
CABG: Coronary artery bypass graft
PCI: Percutaneous coronary intervention
ACS: Acute coronary syndrome
HRT: Hormone replacement therapy
MI: Myocardial infarction
STEMI: ST-elevation myocardial infarction
Non STEMI: Non ST-elevation myocardial infarction
HLP: Hyperlipidemia
